# Chemical Changes in Layered Ferroelectric Semiconductors Induced by Helium Ion Beam

**DOI:** 10.1038/s41598-017-16949-3

**Published:** 2017-11-30

**Authors:** Alex Belianinov, Matthew J. Burch, Holland E. Hysmith, Anton V. Ievlev, Vighter Iberi, Michael A. Susner, Michael A. McGuire, Peter Maksymovych, Marius Chyasnavichyus, Stephen Jesse, Olga S. Ovchinnikova

**Affiliations:** 1 The Institute for Functional Imaging of Materials, Oak Ridge National Laboratory, Oak Ridge, TN 37831 USA; 2 Center for Nanophase Materials Sciences, Oak Ridge National Laboratory, Oak Ridge, TN 37831 USA; 3 Department of Materials Science and Engineering, University of Tennessee, Knoxville, Knoxville, TN 37996 USA; 4 Materials Sciences and Technology Division, Oak Ridge National Laboratory, Oak Ridge, TN 37831 USA

## Abstract

Multi-material systems interfaced with 2D materials, or entirely new 3D heterostructures can lead to the next generation multi-functional device architectures. Physical and chemical control at the nanoscale is also necessary tailor these materials as functional structures approach physical limit. 2D transition metal thiophosphates (TPS), with a general formulae Cu_1−x_In_1+x/3_P_2_S_6,_ have shown ferroelectric polarization behavior with a *T*_*c*_ above the room temperature, making them attractive candidates for designing both: chemical and physical properties. Our previous studies have demonstrated that ferroic order persists on the surface, and that spinoidal decomposition of ferroelectric and paraelectric phases occurs in non-stoichiometric Cu/In ratio formulations. Here, we discuss the chemical changes induced by helium ion irradiation. We explore the TPS compound library with varying Cu/In ratio, using Helium Ion Microscopy, Atomic Force Microscopy (AFM), and Time of Flight-Secondary Ion Mass Spectrometry (ToF-SIMS). We correlate physical nano- and micro- structures to the helium ion dose, as well as chemical signatures of copper, oxygen and sulfur. Our ToF-SIMS results show that He ion irradiation leads to oxygen penetration into the irradiated areas, and diffuses along the Cu-rich domains to the extent of the stopping distance of the helium ions.

## Introduction

Advances in nanofabrication are the driving force behind technological breakthroughs in performance and miniaturization. Layered materials, in particular, are beginning to take center stage as the next generation components for information technology devices^1–3^. In particular, functional layered materials with dielectric properties, such as insulators, semiconductors, and correlated electron materials will be necessary for fabrication and delivery of this new age technology^4–7^.

Resist-based lithography is currently the mainstay of nanostructure fabrication, however, resist free nanofabrication directly on the surface is bringing electron, ion, and probe technologies to the forefront^8–10^. Helium Ion Microscope (HIM)^11,12^, is a particularly attractive tool for nano-patterning of 2D materials *in-situ*, and is being explored by a growing number of groups world-wide^13–18^. Helium (and Neon) ion beam nanofabrication has the benefits of resist-free processing; shorter penetration depth into the bulk, and reduced parasitic ion implantation – a common issue in gallium based Focused Ion Beam (FIB) applications^11,19^. The use of a helium ion microscope (HIM) has already been demonstrated for graphene, MoSe_2_, WSe_2_ and MoS_2_, through controlled ion dose exposure, and tuning of the local defect chemistry; changing the properties of these materials^15,16,20–22^.

We demonstrate HIM to create a variety of micro-sized structures with varying chemical properties atop several transition metal thiophosphates (TPS); a broad class of van-der-Waals layered solids^10,13,23^. TPS exhibit strong ionic character in the chemical bonds between transition metals and the P_2_S_6_ framework^24^. Furthermore, these compounds have large band-gaps and a pronounced flexibility in ionic substitution of the metal sites; with over 260 combinations known for the sulfides alone. Some of these compounds support coherent ordering of metal ions across layers, with ferroelectric and anitferroelectric ground states^14,15^, dielectric relaxor behavior^16,17^, and associated phase transitions. A combination of experimental techniques revealed that the Cu_1−x_In_1+x/3_P_2_S_6_ system undergoes chemical phase separation into centrosymmetric In_4/3_P_2_S_6_ and ferroelectric CuInP_2_S_6_ while maintaining layered framework of the parent compound. The structural coherence of the decomposition enables the increase of the ferroelectric *T*_*c*_ to 335 K, making Cu-deficient Cu_1−x_In_1+x/3_P_2_S_6_ the highest temperature layered, van-der-Waals gapped ferroelectric material yet known^25^.

Our earlier work demonstrates that by controlling the helium dose on a CuInP_2_S_6_ with a 1:1 stoichiometric ratio of Cu/In yields a loss in ferroelectricity, and growth of conical structures that scale in volume with the ion dose^18,26^. However, the nature of the growth process and the exact chemical composition of the ensuing nanostructures remained a mystery. In this work, we explore the helium ion surface interaction within a library of the TPS materials, Cu_1−x_In_1+x/3_P_2_S_6_, with the adjusted Cu ratios ranging from 5 to 100% in order to elucidate their chemical structure. We use Atomic Force Microscopy (AFM), to check the veracity of micro- and nano- sized structures, as well as Secondary Ion Mass Spectrometry (SIMS) to track the chemical changes in the helium irradiated area. Our results demonstrate that copper concentration dictates the sizes of the nanostructures, as the same helium dose applied to the same area yields different volumes in the final protrusions.

## Results

**Figure 1:**
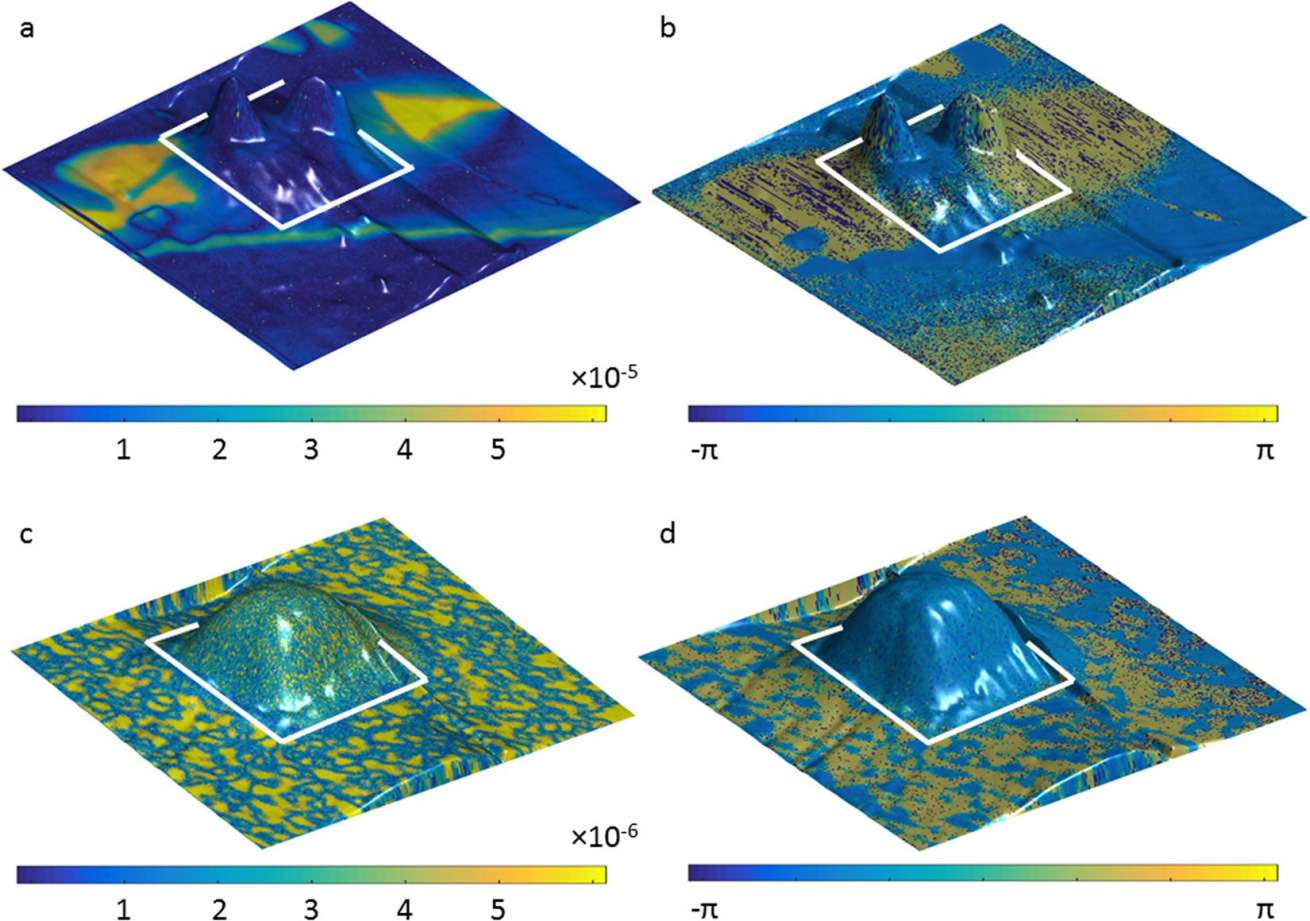
Band Excitation Piezoresponse Force Microscopy (BE-PFM) results. The BE-PFM signal color map, for all figures, is overlaid on the AFM topography. White boxes, in all panels, indicate the HIM irradiated areas. (**a**) The BE-PFM amplitude of the Cu_0.19_ sample; (**b**) The BE-PFM phase of the Cu_0.19_ sample; (**c**) The BE-PFM amplitude of the Cu_0.70_ sample; (**d**) The BE-PFM phase of the Cu_0.70_ sample.

Throughout the article we will use the values for the Cu concentration only, in order to represent different compound formulae, *i.e*. Cu_0.05_ for Cu_0.05_In_1.32_P_2_S_6_. Large, defect-free terraces on TPS were prepared using standard graphene exfoliation techniques. The TPS samples were mounted on conductive carbon tape (PELCO Tabs™, Ted Pella 16084-3) and grounded via stainless steel pucks in AFM and HIM experiments (see Methods section for details). To investigate the effects of the stoichiometric ratio of Cu/In on the growth of the nanostructures, we irradiated the samples with different Cu compositions with an array of He doses. Figure 1(a–d) shows Band Excitation Piezoresponse Force Microscopy^27^ (BE-PFM) amplitude (a, c) and phase (b, d) signals overlaid on the 3D surface topography; the irradiated regions for the Cu_0.19_ Fig. 1(a,b) and Cu_0.70_ Fig. 1(c,d) are highlighted by white boxes. Pure topography images can be found in Suppl. Mat. Fig. S5. Ferroelectric domain networks of the Cu rich phase are shown in yellow in Fig. 1(a,b) for Cu_0.19_ and Fig. 1(c,d) for Cu_0.70_ respectively. Note the sharp domain discontinuity in the Cu_0.19_ samples visible in both the amplitude Fig. 1a and the phase Fig. 1b signals. It is especially well resolved in the phase signal, where the ferroelectric domain (yellow region) is bisected by the ion exposure (blue region) within the white box. The ToF-SIMS results, discussed in more detail below, show that oxygen replaces sulfur ions, which results in the growth of vertical structures within the exposed area. The authors note, that the samples are exposed to atmosphere during the transfer between the HIM and the ToF-SIMS, as well as during the AFM experiments. Furthermore, our HIM mounted residual gas analyzer (Stanford Instruments, RGA 200) indicates the presence of oxygen and water at 1 × 10^−8^ torr and 6 × 10^−8^ torr respectively in the HIM chamber even after a heavy plasma clean. Therefore, oxidation may occur within the HIM chamber immediately as the TPS surface is exposed to the beam. Additionally, as can be seen from Fig. 1a, irradiated areas with the ferroelectrically active Cu phase (yellow in panels a, c) grow wider and taller, as opposed to the paraelectric, In_4/3_P_2_S_6,_ areas shown in blue. The behavior is also similar in Cu_0.70_ illustrated in Fig. 1(b,d) as a large cubic structure. Interestingly, at higher Cu concentrations the entire irradiated area as well as a small percentage of the surrounding area grow isotropically, presumably due to a more consistent Cu dispersion. Earlier ambient and vacuum BE-PFM studies have shown regions of ferroelectric CuInP_2_S_6_ and dielectric In_4/3_P_2_S_6_ coexisting within each layer of the sample^26^, where the ferroelectric phase consisted of continuous domains of oppositely oriented out-of-plane polarization. PFM carried out in this work after modification by HIM showed similar domain structure in the unperturbed areas of the sample (yellow domains in Fig. 1(c,d)).

**Figure 2:**
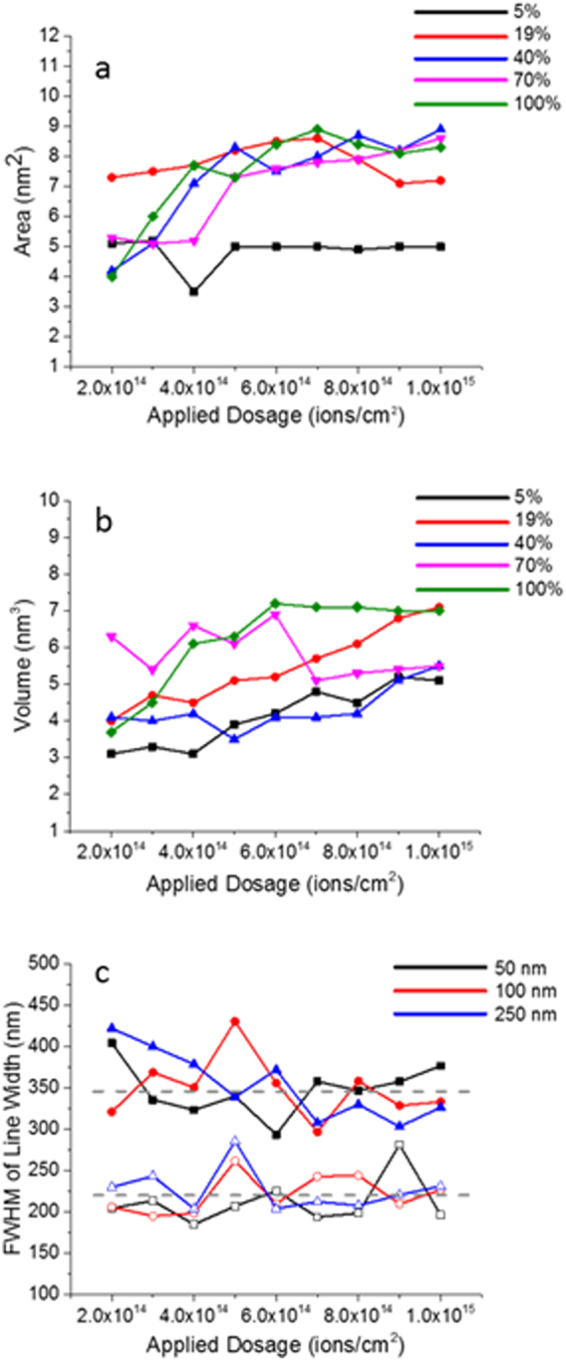
Statistics of the exposed regions on TPS compound library. (**a**) Area of the resulting structures for 1 × 1 um^2^ exposed areas as a function of dose. Different color lines represent different copper concentrations supplemented by a legend in the upper right. (**b**) Volume of the resulting structures for 1 × 1 um^2^ exposed areas as a function of dose. Different color lines represent different copper concentrations supplemented by a legend in the upper right. (**c**) Full Width Half Max (FWHM) of the irradiated lines 1 um long with varying widths as shown by different color lines supplemented by the legend in the upper right. Two sets of samples are presented the low and the high copper concentration the Cu_0.05_ (open symbols) and the Cu_0.7_ (solid symbols).

Figure 2 illustrates the relationship between the area and the volume of modified regions as a function of He-ion dose, and sample composition. We irradiated all samples with rectangular patterns, 1 μm long and 50, 100, 250 nm wide. Unfortunately, since the domain distribution cannot be visualized directly in the HIM, we could not ensure that the nanostructure area is limited to the purely ferroelectric or the dielectric phase. This may be related to the relatively large standard deviation in all plots. On the other hand, for the purpose of creating functional nanostructures, such as interconnects and heterogeneous 2D material stacks; both dielectric and ferroelectric areas are pertinent. Figure 2(a,b) is the area and volume of the resultant structures within the rectangular are exposed to helium irradiation as measured by AFM. The general trend shows increasing area and volume as a function of dose, especially for higher copper concentration samples. Figure 2c is the full width half-max (FWHM) of averaged data for the 50, 100, and 250 nm lines written on Cu_0.05_ (open symbols) and Cu_0.70_ (filled symbols) with doses ranging from 2 × 10^14^ to 1 × 10^15^ ions/cm^2^. In both samples, the widths of the lines do not follow any observable trend with increasing dose, likely due to a mixture of the paraelectric and the ferroelectric phases in the areas of helium exposure. Furthermore, the difference in the smallest possible structures is also probably tied to interaction volume of the helium ions with the bulk material. If we assume a uniform target density of 2.8470 g/cm^3^ based on stoichiometric ratios for Cu_0.70_ the helium ion projected range at 30 keV is 274 nm (calculated by the IONiSE^28^ simulation) which is similar in size to the patterned structures. However, in the copper poor samples such as the Cu_0.05_, the density and penetration depth can vary greatly locally. Therefore, a potential pathway to reduce structure size would be to limit the helium ion interaction with the material by irradiating individual 2D flakes of only a few layers, where the structure size would nominally be determined by the flake thickness.

**Figure 3:**
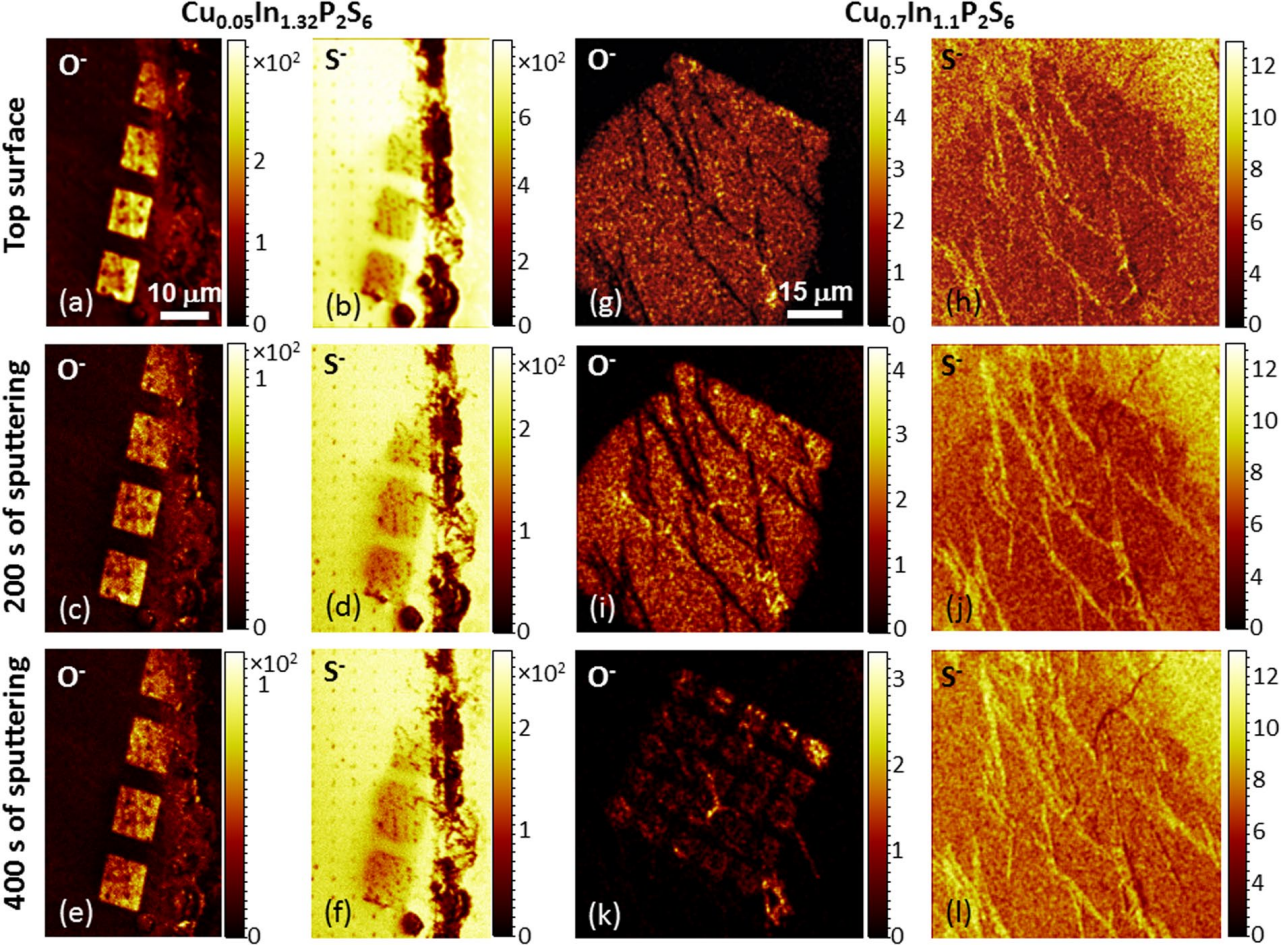
Time of Flight Secondary Ion Mass Spectrometry (ToF-SIMS) results on irradiated helium ion irradiated regions for Cu_0.05_In_1.32_P_2_S_6_ (panels a–f) & Cu_0.7_In_1.1_P_2_S_6_ (panels g–l) (**a**) O^−^ signal for Cu_0.05_In_1.32_P_2_S_6_ at the surface of the sample with no Cs sputtering; total ion count 1.97 × 10^6^ (**b**) S^−^ signal for Cu_0.05_In_1.32_P_2_S_6_ at the surface of the sample with no Cs sputtering; total ion count 2.52 × 10^7^ (**c**) O^−^ signal for Cu_0.05_In_1.32_P_2_S_6_ at the surface of the sample after 200 s of Cs sputtering; total ion count 6.72 × 10^5^ (**d**) S^−^ signal for Cu_0.05_In_1.32_P_2_S_6_ at the surface of the sample after 200 s of Cs sputtering; total ion count 7.71 × 10^6^ (**e**) O^−^ signal for Cu_0.05_In_1.32_P_2_S_6_ at the surface of the sample after 400 s of Cs sputtering; total ion count 6.45 × 10^6^ (**f**) S^−^ signal for Cu_0.05_In_1.32_P_2_S_6_ at the surface of the sample after 400 s of Cs sputtering; total ion count 7.63 × 10^6^ (**g**) O^−^ signal for Cu_0.7_In_1.1_P_2_S_6_ at the surface of the sample with no Cs sputtering; total ion count 5.92 × 10^4^ (**h**) S^−^ signal for Cu_0.7_In_1.1_P_2_S_6_ at the surface of the sample with no Cs sputtering; total ion count 4.40 × 10^5^ (**i**) O^−^ signal for Cu_0.7_In_1.1_P_2_S_6_ at the surface of the sample after 200 s of Cs sputtering; total ion count 5.23 × 10^4^ (**j**) S^−^ signal for Cu_0.7_In_1.1_P_2_S_6_ at the surface of the sample after 200 s of Cs sputtering; total ion count 4.96 × 10^5^ (**k**) O^−^ signal for Cu_0.7_In_1.1_P_2_S_6_ at the surface of the sample after 400 s of Cs sputtering; total ion count 1.15 × 10^4^ (**l**) S^−^ signal for Cu_0.7_In_1.1_P_2_S_6_ at the surface of the sample after 400 s of Cs sputtering; total ion count 5.32 × 10^5^.

To investigate the chemical composition of the nanostructures we carried out time of flight secondary ion mass spectrometry (ToF-SIMS) measurements, with an IONTOF SIMS.5 instrument using a Bismuth primary source and a Cesium sputtering source. ToF-SIMS measurements (Fig. 3, Supplementary Figs S1 and S2) have shown significant amounts of oxygen on the surface and in the bulk of irradiated regions (Fig. 3(a,c,e and g,i,k), which suggests the origin of these changes is the He-ion irradiation. Intensity signal profiles for the sulfur and oxygen signal are shown in Suppl. Mat. Figs S3 and S4. Furthermore, ToF-SIMS results show a decrease in the sulfur concentration near irradiated regions, seen as a halo in the S^−^ signal, Fig. 3(b,d,f and h,j,l). Detailed analysis of the ToF-SIMS data revealed a significant difference in the oxidation levels of the samples with different compositions. Sample with lower Cu concentration (Cu_0.05_; Fig. 3(a–f)) show deeper penetration of oxygen into the irradiated squares (Fig. 3(a,c,e)). While in the samples with higher Cu concentration, (Cu_0.70_ Fig. 3(g–l)) the depth of the oxygen penetration was much smaller (Fig. 3g,i,k). Although, ToF-SIMS measurements allow bulk measurements, we were not able to quantitatively measure the depth of ToF-SIMS imaging, as the sputtering rates in these complex multicomponent samples are difficult to estimate. However, since the density of both samples is relatively close, we can qualitatively compare the depth of oxygen penetration. In the Cu_0.05_ samples, oxygen levels were elevated after 1500 seconds of sputtering. Whereas in the Cu_0.70_ samples, oxygen disappeared after about 400 seconds of sputtering. Interestingly, the Cu_0.70_ sample showed significant lateral oxidation outside of the irradiated regions. This hints at the kinetics of the oxidation process, which are likely to be two-step with a (i) quick lattice destruction, followed by (ii) slower oxygen diffusion. SIMS imaging beyond the surface layer, shows oxygen inside irradiated squares only, (Fig. 3f) however, these SIMS images are taken after 400 s of Cs gun sputtering, penetrating well within the sample bulk, and illustrate oxygen signal spreading laterally outside the irradiated region (Fig. 3e,k). The observed oxygen depth penetration difference between the two compositions could also be responsible for the difference in the structure size observed in Fig. 2 since oxidation seems to be the dominant chemical change after the irradiation process.

## Discussion

In summary, we have explored the effect of helium ion irradiation on the surfaces of a library of TPS compounds using AFM and ToF-SIMS. After the helium exposure, we have used the AFM to observe surface nano- and micro- structures that scale in area and volume, to the total dose of the helium ion beam, as well as the overall copper concentration in the sample. Furthermore, our ToF-SIMS results show that after ion irradiation, the oxygen concentration in the irradiated areas is significantly increased. In samples with lower Cu concentration the oxygen penetration was higher than in samples with more copper. Finally, nanostructured patterns, consisting of lines 50, 100, and 250 nm wide and 1 µm long, atop the library of TPS compounds with varying copper concentrations, reveal a general trend on decreasing structure veracity as the copper concentration is increased.

## Methods

### Sample Synthesis

TPS crystals were grown by chemical vapor transport in evacuated quartz ampoules. The In_2_S_3_ precursor was prepared from Indium (Alfa Aesar Puratronic 99.999%) and Sulphur (JMC Puratronic 99.999%) by reacting them in an appropriate stoichiometric mixture at 950 °C for 48 hrs. In_2_S_3_ product was ground into powder, and checked for purity by using X-ray diffraction. We then added the necessary quantities of Cu, P, and S (Alfa Aesar, Puratronic 99.999%+) to obtain the compositions tested. We combined these precursors in a mortar and pestle in a He-filled glovebox, loaded the powder into quartz ampoules, and sealed under vacuum. The ampoules were brought to 750–775 °C over a period of 15 hrs in a box furnace, held at temperature for 96 hrs., and then cooled at a rate of 20 °C/hr. to 30 °C. After cooling, the crystals were extracted by slicing open the ampoules. The products appear as platelets ranging in area from 2 to 1 mm^2^ and approximately <150 μm thick. TPS samples were mounted on to metal disks (Ted Pella Prod. No. 16218) with a conductive carbon tape; with a grounding wire attached directly to the disk. A clean surface was prepared by “Scotch tape method” widely used for graphene preparation^29^.

### Ambient Imaging

Ambient Band Excitation Imaging was done on a Cypher AFM ES manufactured Asylum Research. Pt-Cr coated Multi-75EG AFM cantilevers (purchased from NanoAndMore) have been used for imaging all samples. For contact mode PFM and topography measurements a setpoint of 0.75–1 V was used. All Band Excitation images were collected at frequency ranges around the third excitation mode of the cantilever (300–400 kHz), using the IgorPro control software as well as in house National Instruments based hardware and software; additional image post-processed was done in WsXM^30^, and Matlab.

### Helium-Ion Microscopy

Scanning helium-ion microscopy measurements were done on a Zeiss ORION Nanofab He+/Ne+ microscope, at 20 kV accelerating voltage, beam current of ~3.0 pA, and chamber base pressure of 1.5 × 10^−7^ mTorr. The sample was irradiated with a He+ beam with dwell time of 0.5 µs and 10 × 10 nm pixel spacing, the ion dose was varied from 1 × 10^14^ to 1 × 10^18^ ions/cm^2^. To minimize ion beam damage and enable subsequent identification of the irradiated regions of interest we acquired low magnification secondary electron images of the nanostructured regions.

## Time of flight Secondary Ion Mass Spectroscopy

ToF SIMS measurements were performed in positive ions detection mode using TOF SIMS-5 (ION-TOF GmbH, Germany) using a 30 keV Bi ion gun as the primary source with a focused ion beam spot size of ~120 nm, current ~500 nA and a *m/*Δ*m* resolution of 60 ÷ 200. The images were collected with a resolution of 256 × 256 points across 80 and 60 µm imaging areas. Depth profiling of the sample was carried out using a Cs^+^ ion-sputtering source operated at 1 keV and 60 nA, and sputtering for 5 ÷ 10 s per slice over a 200 µm total area. The data was collected in negative ions detection mode.

## Electronic supplementary material

Supplementary Information
