# Supplementary material for: Chemical Changes in Layered Ferroelectric Semiconductors Induced by Helium Ion Beam
Source: Sci Rep. 2017 Nov 30;7:16619. doi: 10.1038/s41598-017-16949-3 (PMC5709364; doi:10.1038/s41598-017-16949-3)
Supplement: Supplementary file 1 — Supplementary Information [file 41598_2017_16949_MOESM1_ESM.pdf]

Supplemental Material for: Chemical Changes in Layered Ferroelectric Semiconductors Induced by Helium Ion Beam

Alex Belianinov,[1,2] Matthew J. Burch,[1,2] Holland Hysmith,[2] Anton V. Ievlev,[1,2] Vighter Iberi,[2,3] Michael A. Susner, [4] Michael A. McGuire,[4] Peter Maksymovych, [1,2] Marius Chyasnavichyus,[1,2] Stephen Jesse,[1,2]
Olga S. Ovchinnikova[1,2*]

1 The Institute for Functional Imaging of Materials, Oak Ridge National Laboratory, Oak Ridge, TN 37831

2. Center for Nanophase Materials Sciences, Oak Ridge National Laboratory, Oak Ridge, TN 37831

3. Department of Materials Science and Engineering, University of Tennessee, Knoxville, Knoxville TN 37996

4. Materials Sciences and Technology Division, Oak Ridge National Laboratory, Oak Ridge, TN 37831

This supplemental information addendum contains additional ToF-SIMS results on the exposed TPS samples. Figure S1 shows the penetration depth of oxygen after helium ion exposure. In Figure S1a, b the penetration is shown after 1500 seconds of material sputtering; In Figure S1c, d the penetration is shown after 400 seconds of sputtering. Figure S2 illustrated the Cu and the In SIMS signals in the helium exposed areas. Figure S3 and Figure S4 illustrate the milling profile for O and S signals as a function of sputtering time. Both figures contrast the signal for the exposed and the unexposed areas. Finally, Figure S5 illustrates standalone AFM topography signal where BE-PFM results were overlain in main text Figure 1.

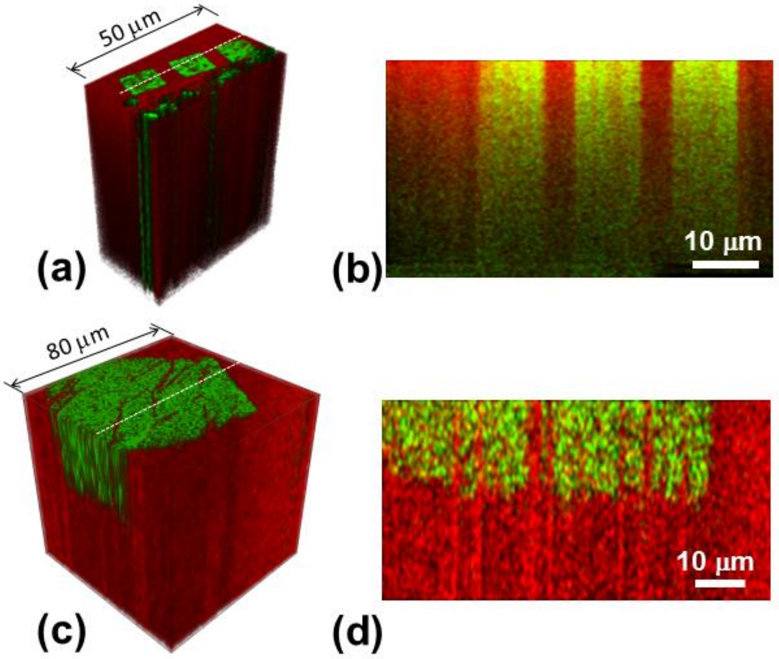

Figure S1. Depth profiling 3D visualization. (a) 3D visualization of the ToF-SIMS results for $Cu_{0.05}In_{1.32}P_2S_6$ where the S^- (red) and the O^- (green) signals are shown; a total of 1500s of sputtering was utilized. (b) X-Z cross-section overlay of S^- (red) and O^- (green) signals for the $Cu_{0.05}In_{1.32}P_2S_6$ after 1500s of sputtering. (c) 3D visualization of the ToF-SIMS results for $Cu_{0.7}In_{1.1}P_2S_6$ where the S^- (red) and the O^- (green) signals are shown; a total of 400s of sputtering was utilized. (d) X-Z cross-section overlay of S^- (red) and O^- (green) signals for the $Cu_{0.7}In_{1.1}P_2S_6$ after 400s of sputtering.

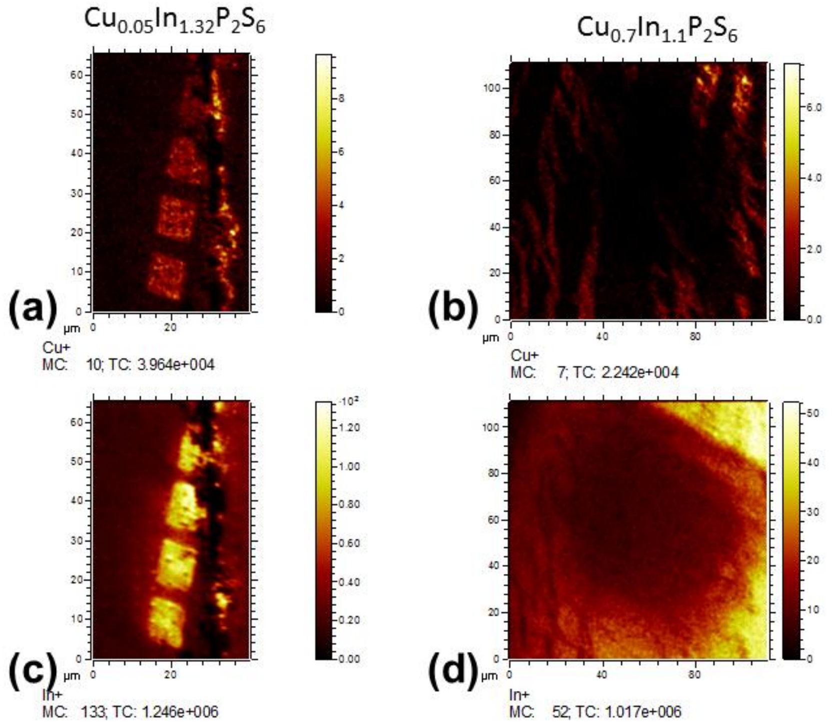

Figure S2. ToF-SIMS results. (a) Distribution of Cu^+ in the irradiated region of $Cu_{0.05}In_{1.32}P_2S_6$. (b) Distribution of Cu^+ in the irradiated region of $Cu_{0.7}In_{1.1}P_2S_6$. (c) Distribution of In^+ in the irradiated region of $Cu_{0.05}In_{1.32}P_2S_{6.}$ (d) Distribution of In^+ in the irradiated region of $Cu_{0.7}In_{1.1}P_2S_6$

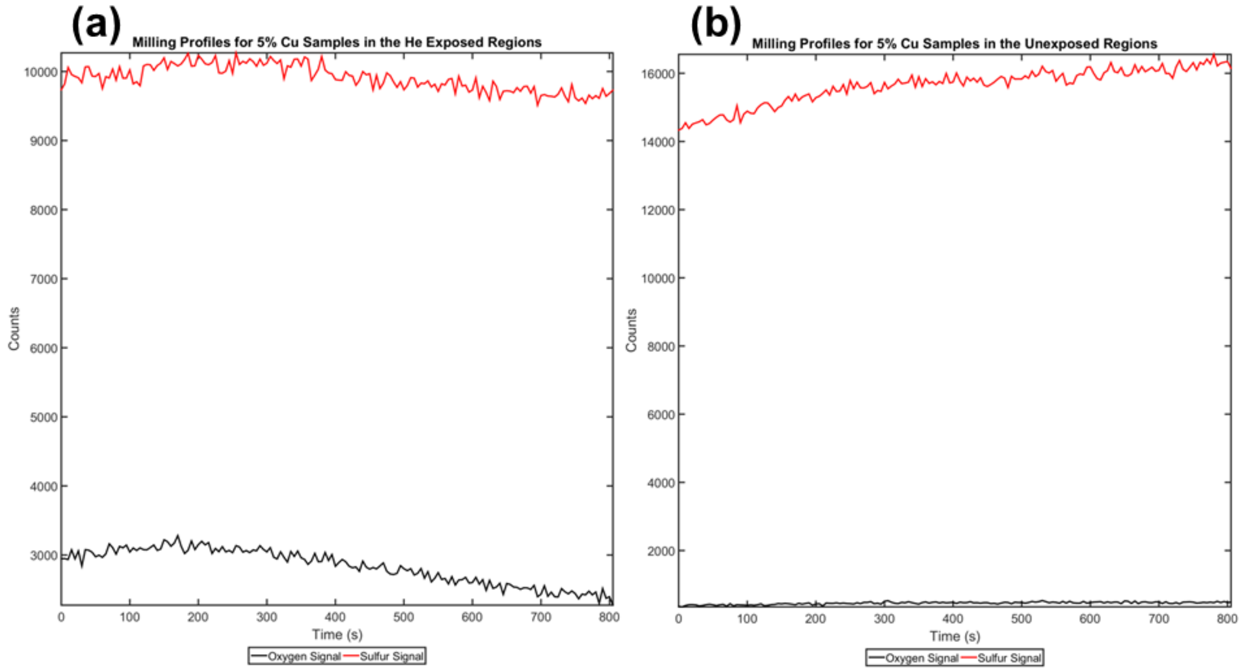

Figure S3. Intensity signal profiling for Oxygen (black line) and Sulfur (red line) in a $Cu_{0.05}In_{1.32}P_2S_6$. (a) Oxygen and Sulfur intensity profiles after 800s of sputtering in the He exposed region. (b) Oxygen and Sulfur intensity profiles after 800s of sputtering in the pristine region.

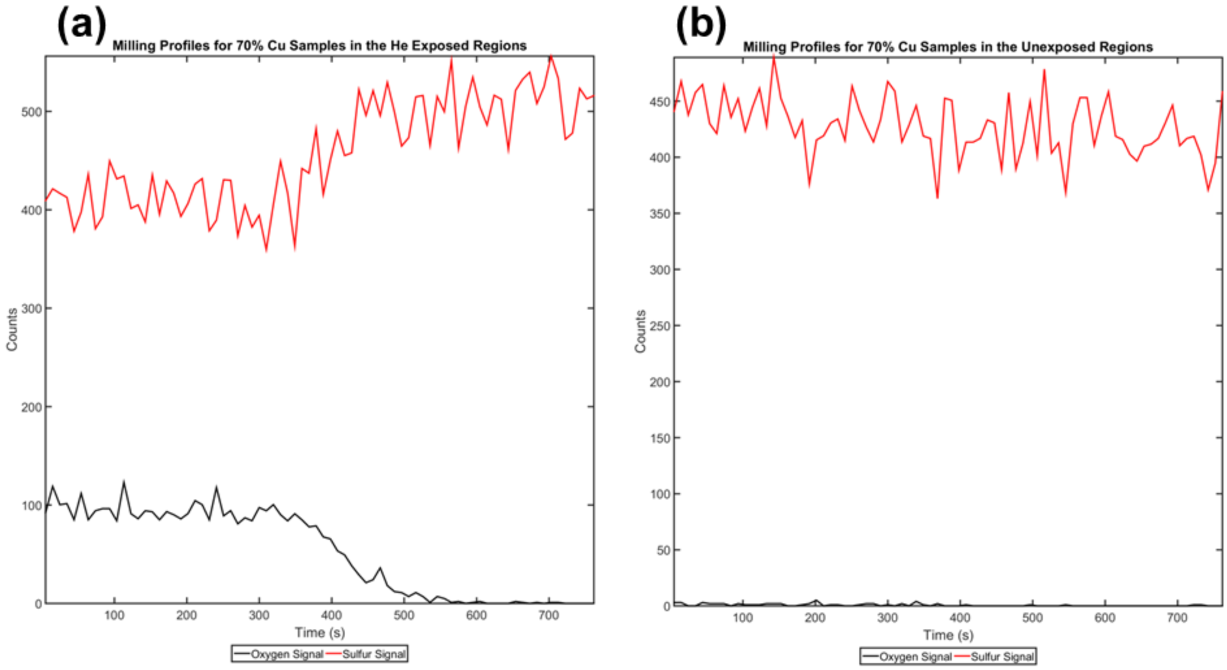

Figure S4. Intensity signal profiling for Oxygen (black line) and Sulfur (red line) in a $Cu_{0.7}In_{1.1}P_2S_6$.(a) Oxygen and Sulfur intensity profiles after 800s of sputtering in the He exposed region. (b) Oxygen and Sulfur intensity profiles after 800s of sputtering in the pristine region.

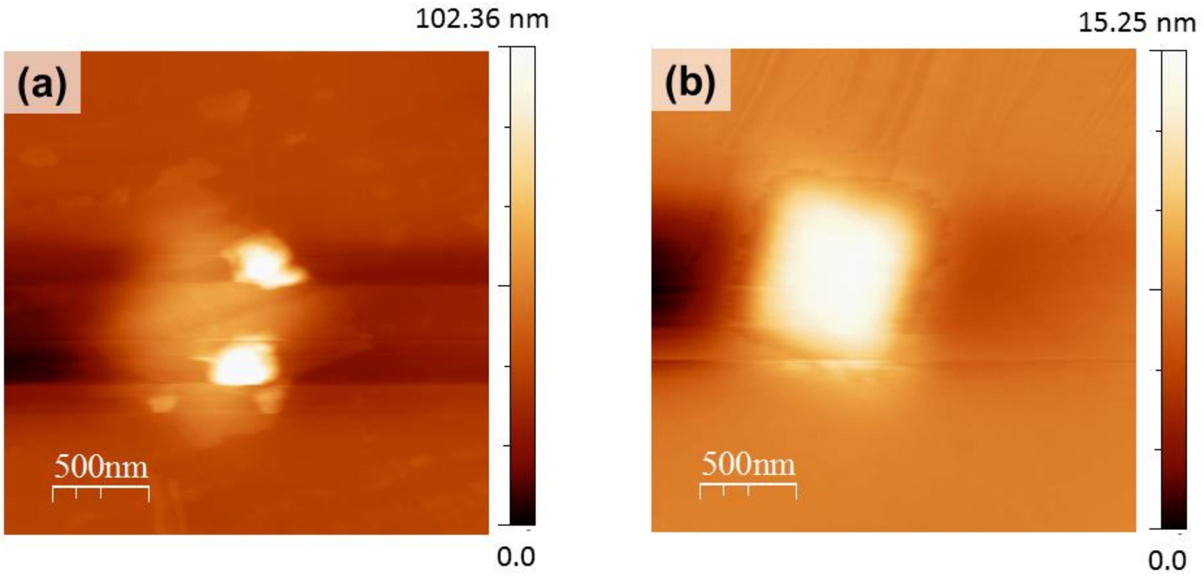

Figure S5. AFM contact mode topography of the 19% Cu and 70% Cu samples after He exposure that were overlaid with the Band Excitation signal in main text Figure 1. (a) Topography for the 19% Cu sample. (b) Topography for the 70% Cu sample.
